# A randomised, double‐blind, placebo‐controlled study to determine the analgesic efficacy, safety and tolerability of VPX638 administered topically to painful wounds

**DOI:** 10.1111/wrr.70008

**Published:** 2025-02-12

**Authors:** Jonathan Golledge, Sergio Parra, Pat M. Aldons, Nicoletta Frescos, Rebecca K. Iseli, Toni M. Pardey, Casper F. Pretorius, Omar R. Shum, Paul A. Yates, Cécile B. Bascoul, Dannette K. Doolittle, Ajay A. Rege, Vaidehi J. Thanawala, Heather Giles, Michael C. Woodward

**Affiliations:** ^1^ Queensland Research Centre for Peripheral Vascular Disease James Cook University and Townsville University Hospital and Australian Institute of Tropical Health and Medicine Townsville Queensland Australia; ^2^ Vapogenix Houston Texas USA; ^3^ The Prince Charles Hospital Brisbane Queensland Australia; ^4^ Austin Hospital University of Melbourne Melbourne Victoria Australia; ^5^ The Royal Melbourne Hospital Melbourne Victoria Australia; ^6^ Novatrials Newcastle New South Wales Australia; ^7^ Mackay Base Hospital Mackay Queensland Australia; ^8^ Wollongong Hospital Wollongong New South Wales Australia

**Keywords:** analgesia, pain, sevoflurane, topical, wound

## Abstract

New analgesics are needed for painful wounds. Multiple reports suggest that topical sevoflurane may have analgesic effects. This placebo‐controlled randomised trial evaluated the analgesic efficacy and safety of VPX638 (topical sevoflurane). Seventy‐eight participants with painful wounds, were enrolled at eight Australian centres and randomly allocated to receive 2 × 5 mL of VPX638 (*N* = 39) or placebo (*N* = 40) during one wound dressing change. Numerical pain rating scores and use of opioids were recorded for 24 h. The primary endpoint was pain during wound cleaning, secondary endpoints evaluated pain for 24 h after drug application and opioids use. There was no significant difference in mean pain scores during wound cleaning between VPX638 and placebo (0.854; *p* = 0.23). The mean differences in summed pain intensity difference from baseline suggested VPX638 provided greater analgesia compared to placebo over 8 h (*p* < 0.02), 12 h (*p* < 0.01) and 24 h (*p* < 0.05) and significantly longer duration of analgesia, 24.3 h for VPX638 versus 7.1 h for placebo (*p* < 0.01). In the 24 h after drug administration, participants receiving VPX638 had a 50% decrease in opioid use over 24 h compared with placebo. VPX638 appeared safe and well‐tolerated. In conclusion, this small placebo‐controlled randomised trial suggested that VPX638 provides analgesia and is opioid‐sparing for up to 24 h after wound cleaning. It supports the need for further evaluation of the benefit of VPX638 as a topical analgesic for painful wounds.

**Abbreviations**
AEs: adverse events
ALT: ALanune Transanimase
AST: ASpartate Transferase
BMI: body mass index
FV: follow‐up visit
ITT: intent‐to‐treat
Kg: kilograms
LFTs: liver function tests
M: metres
mITT: modified ITT
MME: Morphine Milligram Equivalents
NRS: Numerical Rating Scale
PIDs: pain intensity differences
PT: preferred term
SD: standard deviation
SEM: standard error of the mean
SOC: system organ class
SPID: summed pain intensity difference
SV: screening visit
TEAEs: treatment‐emergent adverse events
TV: treatment visit

## 1 INTRODUCTION

Worldwide approximately 400 million people suffer from wounds.^1^ Of these, more than 60 million have chronic wounds such as venous, ischemic and neuropathic lower extremity ulcers.^1^, ^2^ The socioeconomic burden of wounds is rising in association with the ageing population and the diabetes epidemic.^3^ The cost of caring for wounds in the US exceeds $50 billion annually.^4^

More than 80% of people with wounds suffer some degree of pain and in most cases this is severe.^5^, ^6^ Pain from wounds is frequently inadequately treated, may become chronic and has been associated with reduced quality of life and increased mortality.^7^, ^8^, ^9^, ^10^ Pain can be experienced at rest, when moving, and during wound care, making management challenging because both longer‐term and acute control of pain is needed.^7^, ^11^ Oral analgesics, including opioids, frequently fail to provide adequate pain relief, particularly during wound care, and have a large range of adverse effects, such as respiratory depression, nausea, constipation and dependancy.^7^ There is an unmet need for a safe and efficacious wound analgesic. The ideal product would be easy to apply, be locally acting, have rapid onset, provide effective analgesia for wound care procedures such as debridement and dressing changes and provide long‐lasting analgesia. Topical analgesics applied directly to the wound could reduce or even eliminate systemic side‐effects and offer an ideal solution for wound pain management. There are no currently available topical analgesics approved for regular use on wounds.

VPX638 is the volatile anaesthetic sevoflurane, a non‐flammable liquid that has been approved for use as an inhaled general anaesthetic administered by vaporisation. Since approval, sevoflurane has been widely used for general anaesthesia but never been developed for alternative applications.^12^ Multiple previous case reports have suggested that sevoflurane has analgesic effects when applied topically to painful wounds.^13^, ^14^, ^15^, ^16^, ^17^, ^18^, ^19^ Sevoflurane may have an analgesic effect due to its interaction with sodium,^20^, ^21^ potassium,^22^, ^23^, ^24^, ^25^ calcium,^26^, ^27^ Hyperpolarisation Activated Cyclic Nucleotide Gated Potassium Channel 1^28^ and other channels,^29^, ^30^ which inhibit the generation of action potentials in peripheral sensory nerve endings.

Multiple case reports and cohort studies have reported successfully using topical sevoflurane as an analgesic for painful wounds, but no prior controlled clinical trial testing the value of this treatment has been published.^13^, ^14^, ^15^, ^16^, ^17^, ^18^, ^19^ Here, we report the first placebo‐controlled, randomised, double‐blind clinical trial evaluating the efficacy of topical administration of VPX638 to painful wounds during wound care procedures. The primary aim was to test the effect of VPX638 on pain during wound care as compared to placebo. Secondary objectives were to assess the effect of VPX638 compared to placebo on wound pain and the use of other analgesics over 24 h, and to determine the safety and tolerability of topical VPX638. Finally, it was anticipated that the outcomes from this first proof‐of‐concept study would inform the design of subsequent Phase 3 pivotal clinical studies.

## 2 MATERIALS AND METHODS

### 2.1 Study design

This randomised, double‐blind, parallel‐group, placebo‐controlled study was conducted at eight sites in Australia from December 2017 to November 2018. The study protocol complied with the ethical rules for human experimentation that are stated in the 1975 Declaration of Helsinki and was approved by the Human Research Ethics Committees for public (Austin Health) and private (Bellberry Limited) health care centres. All participants included in this study provided written informed consent. Prior to first patient enrolment, the trial was registered in the Australian New Zealand Clinical Trials Registry (registration number: ACTRN12617001629325). Study sites were specialist wound clinics, in‐patient vascular sites or clinical study centres. The study was reported following the CONSORT guidelines for randomised controlled trials.^31^ Wound pain was evaluated using a Numerical Rating Scale (NRS) from 0 to 10, where 0 represented ‘no pain’ and 10 represented the ‘worst pain you can imagine’.

### 2.2 Participants

Eligible participants were ≥18 years with at least one painful wound of size (area) ≤100 cm^2^, which had been open for at least 14 days and with wound pain at rest or at any time during the wound care procedure at the screening visit rated as 5 or greater on the NRS. All wound types, except burns, were eligible. The study excluded participants with a history or family history of a life‐threatening reaction to general anaesthesia, or wounds on the face, head, or neck. Other key exclusion criteria included laboratory abnormalities indicating severe anaemia, leukopenia, liver injury, or kidney disease. Those with any serious medical condition or any other factor which compromised participation or follow‐up in the study, and any recent change (within 2 weeks prior to the treatment visit) in prescribed pain medication with the potential to confound pain assessments were also excluded.

### 2.3 Study drug

The investigational drug was VPX638. VPX638 is topical sevoflurane (Fluoromethyl 2,2,2‐trifluoro‐1‐(trifluoromethyl) ethyl ether). The placebo was 0.9% sodium chloride. VPX638 and placebo were manufactured and packaged for Vapogenix under Good Manufacturing Practice (GMP) requirements in single use 12‐mL amber glass vials.

### 2.4 Randomisation

Study drug vials were labelled by the manufacturer according to a randomised master vial list generated by a third‐party statistician. At the treatment visit eligible consenting participants were stratified according to rest pain score (<5 or ≥5), wound type (venous, neuropathic ulcer or other) and study site. They were then randomised in a 1:1 ratio to VPX638 or placebo, through an independently run interactive web‐based randomisation system using a block size of four which minimised any assignment imbalances. Investigators, study participants, all study site and data management personnel were blinded to treatment group assignment. Treatment unblinding for the study occurred after all clinical data had been received, data inconsistencies and queries had been resolved, and the database locked.

### 2.5 Interventions

Wound care prior to drug administration was at the discretion of the treating physician. Study drug was applied twice during a single visit as follows.

#### 2.5.1 First drug application (D1)

Following dressing removal, 5 mL of study drug was applied to the wound, and additionally to a gauze pad if the wound did not have the volume to contain all the study drug. Immediately after drug application, the gauze pad was applied to the wound and the wound was wrapped in plastic wrap for 15 min. Pain assessments were made at 5, 10 and 15 min after the first administration of study drug. Wound care procedures, as determined by physician assessment, commenced 15–20 min after first drug application. Pain assessments were made following cleansing and/or debridement to document the pain experienced during the procedure.

#### 2.5.2 Second drug application (D2)

A second 5 mL of study drug was administered to the wound immediately prior to application of physician‐dictated wound dressings. Pain assessments were made immediately before the second administration of study drug, and then at 30 min and 1 h after the second drug administration. Participants remained under observation for at least 1 h after the second drug application and not permitted to drive or use heavy machinery for 12 h.

### 2.6 Outcomes

Pain assessments were conducted by trial coordinators at rest (prior to dressing removal), immediately after dressing removal and immediately prior to first drug application (baseline). Wound pain was also assessed at 2, 4, 6, 8, 12, 18 and 24 h after drug administration via participant completed diary cards. These cards were also used to record analgesic use. Participants were also followed up at 3–7 days after drug application, to assess adverse events, liver function tests (LFTs) and the wound.

### 2.7 Pre‐specified outcome measures

The primary efficacy endpoint was the pain during the wound care procedure assessed by comparison of mean NRS scores of participants receiving VPX638 and placebo.

Five key secondary and exploratory efficacy endpoints were evaluated:

- Change in baseline in NRS over 8 and 24 h following first drug application: summed pain intensity difference (SPID_[0‐8h]_ and SPID_[0‐24h]_). The higher the SPID the better the analgesic effect during the specified time period
- Time to onset of analgesic action after the first study drug administration
- Change from baseline in NRS within 15 min after the first study drug administration (SPID_1[0‐15min]_)
- Duration of analgesic effect assessed by time to event Kaplan–Meier methods and Cox proportional hazard analysis
- Use of opioid analgesics in the 24 h following application

#### 2.7.1 Primary endpoint change during study

The original primary endpoint was ‘Change from Baseline in NRS within 15 min after the first study drug administration’. The change was because the original primary analysis population was only to include participants with a baseline rest pain of five or more and at the time of protocol amendment, only 56% of enrolled participants fulfilled this criterion, which was considerably lower than originally anticipated. Therefore, one of the secondary endpoints—pain during wound cleaning—became the new primary endpoint. The change ensured that the maximum number of participants were included in the primary endpoint analysis.

Safety endpoints included the incidence of treatment‐emergent adverse events, change from baseline in alertness level (sedation), vital signs, wound site reactions (erythema, edema, pruritus, urticaria and other), liver function tests, wound size, wound appearance and other adverse events. Treatment‐emergent adverse events were defined as adverse events that occurred or worsened following the first application of study drug. Observational sedation assessments were performed prior to first drug administration, 15 min after first drug application and 30 min and 1 h after second drug application. This was accomplished by using a scale (adapted from two sleepiness scales^32^, ^33^) from 1 to 9, where 1 indicated ‘very alert’ and 9 ‘asleep’.

### 2.8 Sample size

Randomisation of up to 120 participants was planned with up to 60 participants assigned per group (VPX638 and placebo). This sample size provided adequate statistical power for the selected original primary endpoint and some secondary endpoints. The sample size calculation was based on an estimated Cohen effect size of 0.6. The smaller than expected number of participants with a baseline rest pain of five or more, meant that analyses of pre‐specified endpoints in this modified intention‐to‐treat (mITT) population were underpowered. Sample size calculations for the revised primary endpoint (pain during wound cleaning), indicated that a sample size of 70–80 was sufficient to achieve adequate statistical power.

### 2.9 Statistical analyses

A statistical analysis plan describing all pre‐specified analyses was finalised prior to data analysis.

The ITT population was defined as all randomised participants. The mITT population was defined as all randomised participants with a baseline pain score of five or more. The safety population was defined as any participant receiving the treatment after randomisation. This population was used for the analysis of safety parameters. Baseline pain was defined as the NRS immediately prior to first drug application for all pre‐specified analyses. Individual time‐weighted pain intensity differences (PIDs) were calculated by subtracting the pain scores at each time point from the baseline pain scores and multiplied by the time. The SPIDs was then calculated as a sum of the individual time‐weighted PIDs. The revised pre‐specified primary efficacy endpoint analysis was based on the ITT population. The secondary and exploratory endpoint analyses were based on the mITT population, unless specified otherwise. Unless otherwise stated, statistical significance was assessed using a two independent samples *t*‐test. For time‐to‐event data, Kaplan–Meier methods (with calculation of chi‐squared *p*‐value), Log‐rank test and proportional hazards model were used. Analgesic effect was defined as a PID ≥1. Missing evaluations were imputed by the last observation carried forward method if the participant had one or more intermittent missing scores but terminal scores (before and after the missing scores) were available or the baseline observation carried forward method if the participant had one or more missing scores and no further scores have been recorded up to 24 h post the second drug administration. A *p* value ≤0.05 was considered statistically significant.

Morphine Milligram Equivalents (MME) were, in general, calculated using the CDC guidance.

Summaries of adverse events included the incidence by MedDRA preferred term (PT) and system organ class (SOC). The adverse events were also summarised by severity, PT and SOC.

## 3 RESULTS

### 3.1 Participants

**FIGURE 1:**
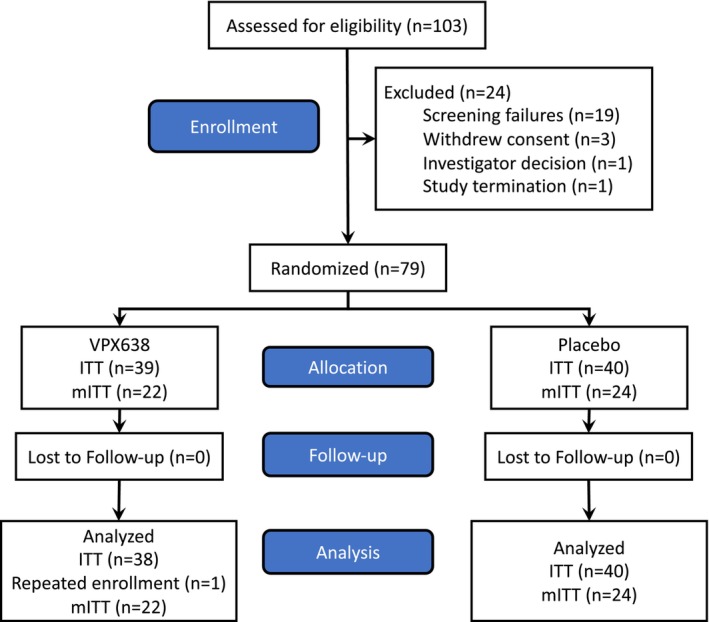
Flow diagram of the study. ITT, intent to treat population; mITT, modified intent to treat population (subjects with a baseline NRS [0–10] pain score at rest of five or more).

Overall, 103 subjects were screened of whom 24 did not meet eligibility criteria and were not randomised. Seventy‐nine participants were randomised from 8 sites in Australia, with 39 assigned to VPX638 and 40 to placebo. The study was stopped for two reasons. First, the required sample size for the revised primary end point was achieved. Second, slow enrolment led to the decision to terminate the study prior to reaching the required sample size for the original primary endpoint. All 79 (100%) participants completed the study and were included in the safety analysis. One participant unexpectedly enrolled in the study twice and this participant's second set of efficacy evaluations were not included in the analyses, resulting in 78 participants (38 assigned to VPX638 and 40 to placebo) being included in the ITT efficacy analyses (Figure 1). Participants demographic data and wound types are shown in Table 1. At baseline, 46 (59%) participants had a pain score of ≥5 just prior to drug application.

**TABLE 1:** Demographics and baseline characteristics.

		VPX638 (*N* = 38)^a^	Placebo (*N* = 40)	Overall (*N* = 78)
Age (years)	Mean (SD)	65.2 (14.7)	68.5 (12.8)	66.9 (13.7)
Gender, *n* (%)^b^	Male	19 (50.0%)	19 (47.5%)	38 (48.7%)
	Female	19 (50.0%)	21 (52.5%)	40 (51.3%)
Race, *n* (%)^b^	Caucasian	37 (97.4%)	39 (97.5%)	76 (97.4%)
	Aboriginal and Torres Strait Islander Peoples	1 (2.6%)	1 (2.5%)	2 (2.6%)
	Other	0	0	0
BMI (kg/m^2^)	Mean (SD)	29.6 (8.7)	34.2 (10.6)	31.9 (9.9)
Wound type, *n* (%)	Venous stasis	17 (44.7%)	19 (47.5%)	36 (46.1%)
	Traumatic/Post‐trauma	8 (21.1%)	3 (7.5%)	11 (14.1%)
	Diabetic neuropathic	3 (7.9%)	5 (12.5%)	8 (10.2%)
	Mixed aetiology	0	7 (17.5%)	7 (9.0%)
	Ischemic or arterial	3 (7.9%)	3 (7.5%)	6 (7.7%)
	Vasculitis	3 (7.9%)	1 (2.5%)	4 (5.1%)
	Infectious	2 (5.3%)	0	2 (2.6%)
	Surgical/Postoperative	1 (2.6%)	1 (2.5%)	2 (2.6%)
	Pressure	1 (2.6%)	0	1 (1.3%)
	Lymphedema	0	1 (2.5%)	1 (1.3%)

Abbreviation: SD, standard deviation.

^a^ Excludes one duplicate participant who was treated at two different sites.

^b^ Self‐reported.

### 3.2 Efficacy endpoints

#### 3.2.1 Pain during wound care procedure (ITT population)

The wound care procedure for 69% of the participants was cleaning only; 18% of participants had blunt or sharp wound debridement. The wound care procedure lasted <3 min (overall 2.7 ± 2.6 min; placebo 2.3 ± 1.9 min; VPX638 3.1 ± 3.1 min, non‐significant difference).

**FIGURE 2:**
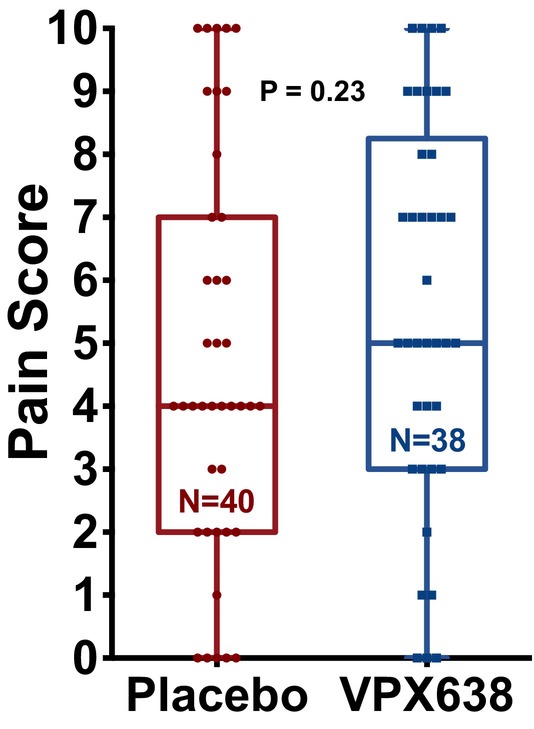
Pain during wound care procedure in the ITT population for VPX638 and placebo groups. Data shown as 25th to 75th percentiles (boxes), min to max (whiskers), and the median (lines in the middle of the boxes). Treatment groups were compared using a two‐sample two‐tailed *t*‐test.

There was no significant difference in the pain during the wound care procedure between the VPX638 and placebo treatment groups when the mean NRS pain score was compared (difference in mean NRS, VPX638 vs. placebo: 0.854; 95% CI: −0.553, 2.261; *p* = 0.231; Figure 2). These results were independent of the type of wound cleaning procedure and recruitment site, although there was a large between‐site difference in the average pain during wound cleaning (NRS varied from 3 to almost 8).

#### 3.2.2 Pain over 15 min following first drug application (mITT)

**FIGURE 3:**
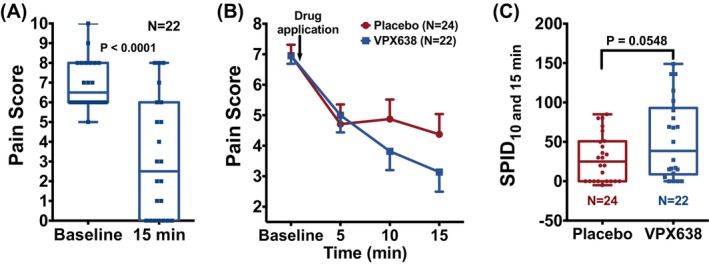
Pain during the first 15 min after study drug application in the mITT population. (A) Comparison of NRS at baseline (0 min) and 15 min after application of VPX638. (B) NRS at baseline (0 min), 5, 10 and 15 min after application of VPX638 or placebo. (C) Post‐hoc analysis: Comparison of SPID over 15 min (without 5‐min time point) of placebo group versus VPX638 group. (A and C) Data shown as 25th to 75th percentiles (boxes), min to max (whiskers), and the median (lines in the middle of the boxes). (B) Data shown as mean ± SEM. Treatment groups were compared using two‐tailed *t*‐tests.

There was a rapid and profound decrease in pain following application of VPX638 (mean decrease in NRS of 3.8 at 15 min, 95% CI: 2.4, 5.2; *p* < 0.0001; Figure 3A). There was an unexpectedly large response in the placebo group at 5 min (Figure 3B) and when the mean SPID_D1[0‐15min]_ were compared, there was no statistical difference between the VPX638 and placebo treatment groups (*p* = 0.408, data not shown).

However, in post‐hoc analysis, removal of the 5‐min time points from the SPID analysis revealed a trend towards a difference between the VPX638 and placebo treatment groups which approached significance (*p* = 0.055; Figure 3C).

#### 3.2.3 Pain during 24 h after drug applications (mITT and ITT)

**FIGURE 4:**
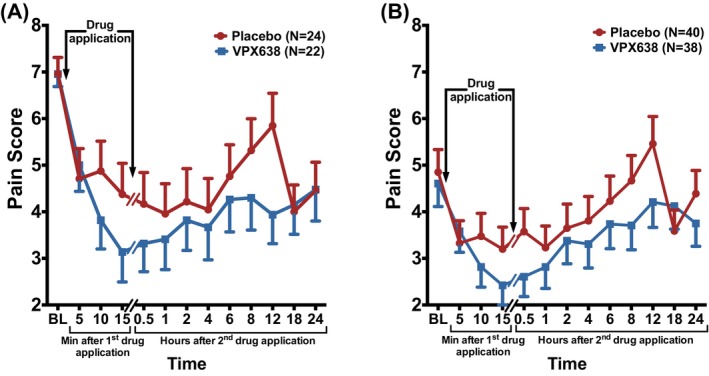
Pain in the mITT (A) and ITT (B) populations for VPX638 and placebo groups over 24 h after study drug application. Data shown as mean NRS ± SEM. BL, baseline.

VPX638 treatment demonstrated greater analgesic effect than placebo at all time points (except 18 h), in both the mITT and ITT populations (Figure 4).

In the pre‐specified analysis, there were no statistically significant differences in SPIDs between the VPX638 and placebo treatment groups for the 0–8 h and the 0–24 h time period, for either the mITT or ITT population.

**FIGURE 5:**
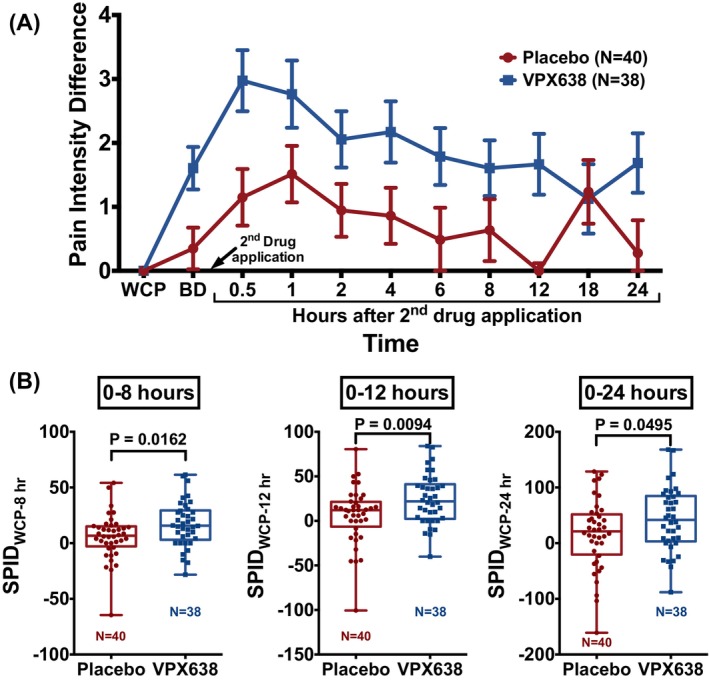
Post‐hoc analysis: PID and SPID in the ITT population for VPX638 (*n* = 38) and placebo (*n* = 40) groups. (A) PID over the 24 h after second drug application. Data shown as mean ± SEM. (B) SPIDs over 8, 12 and 24 h. Data shown as 25th to 75th percentiles (boxes), min to max (whiskers), and the median (lines in the middle of the boxes). Treatment groups were compared using a two‐sample two‐tailed *t*‐test. BD, before drug; WCP, wound care procedure.

Post‐hoc analyses of the ITT population revealed significant differences between the two groups, using pain during the wound care procedure as baseline. Pain intensity difference was greater than placebo in the VPX638 group during the whole 24‐h period (Figure 5A). There were statistically significant differences in SPIDs between the VPX638 and the placebo group over 8 h (11.34; 95% CI: 2.16, 20.52; *p* = 0.0162) and 12 h (18.06; 95% CI: 4.57, 31.55; *p* = 0.0094). A significant difference remained over 24 h (27.07; 95% CI: 0.06, 54.08; *p* = 0.0495) (Figure 5B). Less than 10% of the data was imputed. The distribution of the variables was not significantly different from normal.

#### 3.2.4 Duration of analgesia (ITT)

**FIGURE 6:**
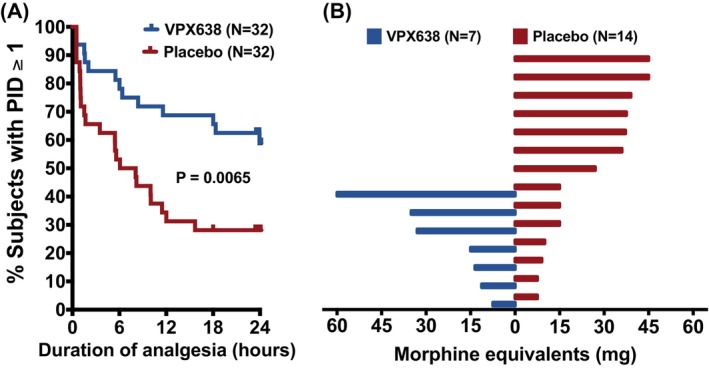
(A) Post‐hoc analysis: Duration of analgesic effect (PID ≥ 1) using NRS during wound cleaning as baseline represented as a Kaplan–Meier plot I the ITT population. Comparison between treatment group curves for VPX638 and placebo used a Log‐rank (Mantel‐Cox) test (*p* = 0.0065). Small vertical lines on the curves indicate censored data. (B) Opioid use for VPX638 and placebo groups. Tornado plot showing per participant total opioid use for 24 h after second drug application.

In post‐hoc analysis, baseline was defined as pain during the wound care procedure. There was a significantly longer median duration of analgesia in the VPX638 group (24.27 h) compared with the placebo group (7.08 h), *p* = 0.0065 (Hazard ratio VPX638/Placebo 0.415, 95% CI: 0.206, 0.765). The Kaplan–Meier plot is shown in Figure 6A.

### 3.3 Concomitant medications and baseline analgesia use

Overall, 78 (98.7%) participants reported using at least one concomitant medication during the study and the 30 days prior to treatment visit. The most common concomitant medications used were in the categories of other analgesics and antipyretics (68 [86.1%] participants) followed by opioids (46 [59%] participants) and antithrombotic agents (41 [51.9%] participants). In general, the treatment groups were balanced with respect to the use of concomitant medications. Of the 59% of participants taking opioids the most common was oral oxycodone (27/46). Median daily opioid use per participant was 23 MME and 13/46 were taking more than 60 mg MMEs daily.

### 3.4 Opioid use after study intervention

Opioid use in the 24 h after drug application was an exploratory endpoint. 46/78 subjects were prescribed and using opioids in the 30 days prior to the treatment visit. Fewer subjects in the VPX638 group (19) were using opioids as compared to the placebo group (27). Of these participants, in the 24 h after second study drug administration, 50% fewer participants took opioids for wound pain in the VPX638 group compared with placebo (7 VPX638; 14 placebo; Figure 6B). There was a similar percentage decrease (53%) when considering opioid use for any reason: (9 VPX638; vs. 17 placebo). Cumulative night‐time opioid use for wound pain decreased by 61% in the VPX638 group. Overall, the VPX638 group had a 44% decrease in daily MME use per participant.

### 3.5 Safety

#### 3.5.1 Overall safety

A total of 28 (35.4%) participants reported at least one adverse event during this study. There were no deaths, serious or severe adverse events or discontinuations. All adverse events were mild or moderate in severity and were assessed as possibly or unlikely related to the study drug. The treatment groups were balanced with respect to the number of participants who experienced at least one adverse event. There were two wound infections reported in the VPX638 group (one in the target wound, one in a different wound) versus 0 in the placebo group. Other wound site adverse events were quite similar (Table 2). There were no clinically meaningful changes in liver function tests observed throughout the course of the study except for one participant who had a history of chronic hepatitis B.

**TABLE 2:** Summary of adverse events.

Preferred term	Number of subjects	
	VPX638 (*N* = 39)	Placebo (*N* = 40)
Nausea	1	0
Wound infection^a^	2	0
Blood potassium increased	1	1
Liver function test abnormal^b^	2	0
Headache	2	1
Erythema	5	5
Edema	6	4
Papules	1	0
Pruritus	4	4
Rash	0	1

^a^ One participant with non‐target wound infection.

^b^ One participant with elevated ALT and AST.

#### 3.5.2 Local wound site reactions

The most common adverse events were local wound site reactions: 21 (26.6%) participants reported 30 wound site adverse events, and their occurrence was similar in the VPX638 and placebo groups (Table 2).

#### 3.5.3 Level of alertness

There was no meaningful change from baseline level of alertness after drug application, and no difference in alertness level between the VPX638 and placebo treatment groups. On average all participants were very alert or alert (scores of 1 or 2) throughout the treatment visit. No other evidence of systemic absorption (e.g., increase in heart rate, headache) was noted.

## 4 DISCUSSION

We conducted the first double blind, placebo‐controlled, randomised multi‐centre clinical trial to evaluate the efficacy and safety of VPX638 (topical sevoflurane) for alleviation of wound pain. Prior to this study, published and unpublished investigator experiences have reported benefit not only for reducing wound rest pain but also in allowing more effective and less painful wound care in the clinic.^13^, ^14^, ^15^, ^16^, ^17^, ^18^, ^19^

The primary efficacy endpoint was the effect of VPX638 on pain during wound cleaning. Adequate wound cleaning, including debridement when necessary, is an important but painful procedure often required to promoting wound healing.^34^, ^35^ It was hypothesised that VPX638 would reduce pain during care and consequently allow improved procedures. Although there was a large and immediate decrease in wound pain following application of topical VPX638 there was no significant difference in pain during wound cleaning between the VPX638 and placebo groups. This was unexpected given multiple previous reports of analgesia.^13^, ^14^, ^15^, ^16^, ^17^, ^18^, ^19^ The absence of analgesia in this study was potentially due to methodological differences. A crucial difference was that only a single application prior to the wound care procedure was allowed in this study, while the previous case reports used multiple applications throughout the procedures. Such an approach was deemed too uncontrolled and variable for this first well‐controlled clinical trial and raised theoretical safety concerns. Additional contributing factors may have been that wound care procedures varied between patients and sites. As is typical in wound studies these aspects were left to the discretion of the treating physician. Therefore, presence of barriers to diffusion such as wound crust and biofilm which could interfere with the analgesic effect of VPX638 were likely to vary considerably. Finally, procedures were terminated when pain reached a certain level, and that level varied between sites. Procedures tended to last 35% longer in the VPX638 group, supporting the possibility that VPX638 allowed better wound cleaning within pain limits. Finally, the type of wound care generally chosen in this study was cleaning and not a more painful debridement procedures used in previous reports.

The current trial experience indicates that additional trials are required to test the hypothesis that topical VPX638 can provide analgesia during wound care procedures. These might include repeat drug application, reduction of between site variation in wound cleaning and by including only participants requiring debridement, not the less painful washing procedures.

Secondary endpoints evaluated the effect of topical VPX638 on rest pain during the 24 h after application of study drug in the mITT population. Fewer than 60% of participants fulfilled the mITT criteria of baseline pain NRS ≥ 5: *n* = 22 and 24 in the VPX638 and placebo groups respectively. Consequently, the sample size for all the pre‐specified rest pain endpoints was lower and thereby not sufficiently powered. This is probably a major reason why none of the pre‐specified rest pain endpoints were significantly different from placebo, even though there was more analgesia in the VPX638 group than in the placebo group at all timepoints except 18 h post application. Therefore, we conducted several post‐hoc analyses, including with the whole ITT population (*n* = 78), in order to fully understand the data.

The immediate, large and clinically meaningful decrease in NRS in the mITT population following VPX638 application was consistent with multiple previous case reports.^13^, ^14^, ^15^ However, there was also a decrease in pain score in the placebo group at the 5 min timepoint which resulted in the pre‐specified analysis showing no statistically significant difference between the groups over the first 15 min. Encouragingly, post‐hoc SPID analysis excluding the 5‐min timepoint did reveal an analgesic effect of VPX638 which very closely approached significance (*p* = 0.0548).

Overall, there was a clear indication that VPX638 resulted in a larger reduction of pain than the placebo beyond that 5‐min time point for approximately 24 h following two applications of drug. Pre‐specified SPID analysis of the mITT population data showed no significant difference at 12, 18 or 24 h after drug application. However, post‐hoc analysis of the larger ITT population, using pain immediately prior to the second drug application as baseline, showed highly statistically significant and clinically meaningful analgesia (compared to placebo) over 12, 18 and even 24 h post drug application. Similar post‐hoc analysis demonstrated a significantly longer duration of action compared with placebo, all providing a strong indication of the analgesic efficacy of topical VPX638.

The large placebo effect throughout the 24 h after drug application resulted in a smaller than predicted effect size—difference between pain score in the VPX638 relative to placebo groups. It is not unusual to see sizable and prolonged placebo effects in clinical trials evaluating analgesic drugs.^36^, ^37^, ^38^, ^39^, ^40^ All the cited studies demonstrated clinically meaningful analgesia with the study drug but the effect size relative to placebo ranged between 0.8 and 1.2 (0–10 NRS scale), similar to that observed in the current study. The small effect size often results in failure to show statistical significance in early clinical development of analgesics. Careful evaluation of such data can result in refinement of clinical design and sample size calculations, and successful entry of new analgesics into clinical use.^36^, ^40^ The reason for large placebo effects is probably multifactorial. The very large reported analgesic response in the placebo group in the first 5 min may have been due to a soothing effect of addition of the 0.9% saline placebo. Expectation bias is a key factor: the participants know they are participating in an analgesia study and expect to feel less pain. In the current study, participants may have anticipated benefit from adding liquid to wound, and from the highly ceremonial drug application process by the presence of impassionate investigators. Study participants often want to ‘please’ site or clinic staff; additionally dressing change and wound cleaning is a highly ‘ritualised’ event where the patient receives a lot of attention. Many of these factors are known to increase the size of the placebo response.^41^, ^42^, ^43^

In the current study multiple study design and analysis strategies were used to counteract the placebo effect and to reduce within patient and site variability in pain reporting, as recommended by a multi‐national advisory working group.^44^, ^45^ These included use of well‐accepted pain measures and analysis methods, extensive regular staff training with a training manual for reference, participant education, standardised scripts and assessment of individual participants by the same outcome assessor throughout the treatment day.

Calculating sample size for the rest pain endpoints was challenging given the absence of any prior published data on the likely effect size and variability, particularly in the placebo group. One of the goals of this first proof‐of‐concept study was to generate the information required to appropriately design and power future wound pain studies, and this has been achieved.

The reduction in the need for, and use of, opioid analgesics in the VPX638 group corroborated the data suggesting that rest pain was reduced in a clinically meaningful way. This effect may have been greater without the well‐known dependency stimulated by opioid drugs. The 67% decrease in overnight opioid use also provided indications that VPX638 improved sleep, presumably due to less pain. There is a recognised unmet need for non‐opioid containing regimens to improve local pain management. This is certainly the case in ulcer management where local pain can be quite significant, the disease course (time to healing) is quite protracted and systemic opioid prescription rates are high^18^, ^46^, ^47^ despite the very considerable societal pressure on physicians to reduce opioid prescribing. In our study, 59% of participants had an opioid prescription at the time of entry to the trial. If the decrease in opioid use is confirmed in a larger cohort of participants in Phase 3 trials, it would represent an important societal benefit.

No concerning safety issues emerged in the study. Notably, safety results were reassuring for two theoretical concerns for participants receiving VPX638. First, there were no signs of clinically meaningful systemic exposure to sevoflurane, with no evidence of sedation, reduced wakefulness, tachycardia or drug related headache. Second, with regards to LFTs, other than a participant with chronic hepatitis B, there were no meaningful increases in aminotransferases and no Hy's law cases. This is consistent with existing case series data which do not report issues related to liver function, even during repeat dosing over several months.^18^, ^48^, ^49^ VPX638 was well‐tolerated locally, both in the wound and the peri‐wound area, with no difference from placebo in local site reactions. Future studies will include repeat dosing of the drug. Sedation, adverse events, LFTs and local tolerance and effect on wound healing will be closely followed parameters.

This study indicates that local application of VPX638 (sevoflurane) has the potential to be an effective and novel means to manage rest pain from open wounds, potentially reducing the need for systemic opioids in the wound care setting. VPX638 treatment was safe and well tolerated in this small, short‐term study. As the first double‐blind placebo‐controlled trial of topical sevoflurane, this trial demonstrated important findings with strong evidence for proof‐of‐concept. In summary, while this trial did not achieve statistical significance in meeting pre‐specified primary and secondary endpoints, it did provide the necessary trial design insights and proof of concept necessary to design endpoints and power appropriately Phase 3 pivotal studies for demonstrating a clinically meaningful reduction in resting pain in a difficult to treat patient population with high unmet medical need for effective, local pain control.

## AUTHOR CONTRIBUTIONS

HG, SP, VT, CB, DD and MW conceived of and designed the study. JG, PA, NF, RI, TMP, CM, CP, OS, PY and MW acquired the data. HG, SP, VT, CB and AR analysed and interpreted the data. HG, SP and JG drafted the manuscript. All authors provided critical revisions to the manuscript and approved its final version.

## CONFLICT OF INTEREST STATEMENT

Vapogenix, Inc. staff received salary and stock options.

## Data Availability

The data supporting this study's findings are available from the corresponding author upon reasonable request. The data are not publicly available due to privacy or ethical restrictions.
